# Beta-blocker management in patients admitted for acute heart failure and reduced ejection fraction: a review and expert consensus opinion

**DOI:** 10.3389/fcvm.2023.1263482

**Published:** 2023-11-16

**Authors:** Guillaume Schurtz, Nathan Mewton, Gilles Lemesle, Clément Delmas, Bruno Levy, Etienne Puymirat, Nadia Aissaoui, Fabrice Bauer, Edouard Gerbaud, Patrick Henry, Laurent Bonello, Thomas Bochaton, Eric Bonnefoy, François Roubille, Nicolas Lamblin

**Affiliations:** ^1^ USIC et Centre Hémodynamique, Institut Coeur Poumon, Centre Hospitalier Universitaire de Lille, Lille, France; ^2^ Hôpital Cardio-Vasculaire Louis Pradel. Filière Insuffisance Cardiaque, Centre D'Investigation Clinique, INSERM 1407. Unité CarMeN, INSERM 1060, Hospices Civils de Lyon, Université Claude Bernard Lyon 1, Lyon, France; ^3^ Institut Pasteur de Lille, Unité INSERM UMR1011, Lille, France; ^4^ Faculté de Médecine de l’Université de Lille, Lille, France; ^5^ FACT (French Alliance for Cardiovascular Trials), Paris, France; ^6^ Intensive Cardiac Care Unit, Rangueil University Hospital, Toulouse, France; ^7^ Service de Réanimation Médicale Brabois, CHRU Nancy, Pôle Cardio-Médico-Chirurgical, Vandoeuvre-les-Nancy, INSERM U1116, Faculté de Médecine, Vandoeuvre-les-Nancy, Université de Lorraine, Nancy, France; ^8^ Department of Cardiology, Assistance Publique des Hôpitaux de Paris, Paris, France; ^9^ Médecine Intensive Réanimation, Cochin, AfterROSC, Assistance Publique-Hôpitaux de Paris (AP-HP), Université Paris Cité, Paris, France; ^10^ Heart Failure Network, Advanced Heart Failure Clinic and Pulmonary Hypertension Department, Cardiac Surgery Department, INSERM U1096, Rouen University Teaching Hospital, Rouen, France; ^11^ Cardiology Intensive Care Unit and Interventional Cardiology, Hôpital Cardiologique du Haut-Lévêque, Pessac, France; ^12^ Bordeaux Cardio-Thoracic Research Centre, INSERM U1045, Bordeaux University, Bordeaux, France; ^13^ Department of Cardiology, Assistance Publique-Hôpitaux de Paris, INSERM U942, University of Paris, Paris, France; ^14^ Cardiology Department, APHM, Mediterranean Association for Research and Studies in Cardiology (MARS Cardio), Centre for CardioVascular and Nutrition Research (C2VN), INSERM 1263, INRA 1260, Aix-Marseille Univ, Marseille, France; ^15^ Intensive Cardiological Care Division, Hospices Civils de Lyon-Hôpital Cardiovasculaire et Pulmonaire, Lyon, France; ^16^ Cardiology Department, INI-CRT, CHU de Montpellier, PhyMedExp, INSERM, CNRS, Université de Montpellier, Montpellier, France; ^17^ Cardiology Department, Heart and Lung Institute, University Hospital of Lille, Lille, France; ^18^ INSERM U1167, Institut Pasteur of Lille, Lille, France

**Keywords:** beta-blocker therapy, acute heart failure, cardiogenic shock, inotropes, left ventricular systolic dysfunction

## Abstract

The role of the beta-adrenergic signaling pathway in heart failure (HF) is pivotal. Early blockade of this pathway with beta-blocker (BB) therapy is recommended as the first-line medication for patients with HF and reduced ejection fraction (HFrEF). Conversely, in patients with severe acute HF (AHF), including those with resolved cardiogenic shock (CS), BB initiation can be hazardous. There are very few data on the management of BB in these situations. The present expert consensus aims to review all published data on the use of BB in patients with severe decompensated AHF, with or without hemodynamic compromise, and proposes an expert-recommended practical algorithm for the prescription and monitoring of BB therapy in critical settings.

## Introduction

Over the past 20 years, randomized trials have made BB therapy an established first-line medication for symptomatic patients with left ventricular (LV) systolic dysfunction (1–4). However, these trials were conducted in stable outpatients, and no solid data are currently available to guide physicians with this therapy in patients with AHF.

To date, physicians continue to be reluctant to initiate, continue, or even optimize BB therapy in the most severe forms of HF (5). The common concern is that hemodynamic destabilization may negatively impact patient outcomes. Nevertheless, this attitude exposes patients to worse long-term prognosis and notably the risk of never having the treatment initiated (6).

Current guidelines are elusive for the management of evidence-base oral medical therapies (OMT, including BB) in patients who experienced a worsening HF episode (with or without hemodynamic instability). Thus, no clear practical guidance is given on when or how physicians should manage BB; this ambiguity has led to substantial heterogeneity in the management of acutely decompensated patients, whether they are already treated with BB or not.

The present review and expert consensus has then two objectives: (1) to summarize current knowledge on BB use in severe decompensated HFrEF, and (2) to propose a pragmatic approach to BB management to assist physicians in their daily practice.

## Pathophysiology of BB therapy in HF

**Figure 1:**
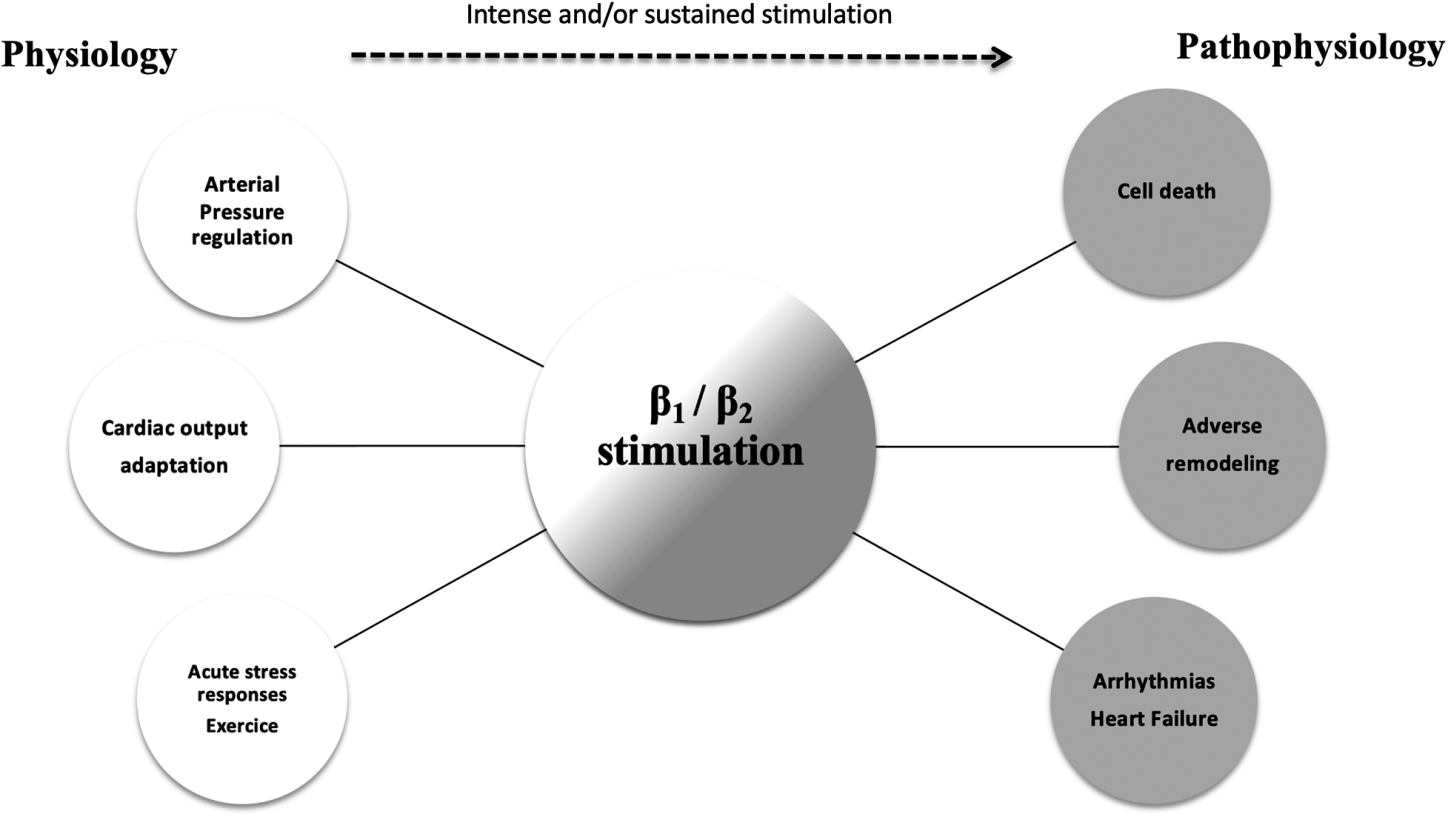
The physiologic and pathophysiologic role of the beta-adrenergic pathway. The role of beta-adrenergic stimulation in cardiac physiology is pivotal (white circles). Intense and sustained stimulation (dotted arrow) are presumed to be responsible for the deleterious effects observed in patients with severe heart failure (gray circles).

The role of the beta-adrenergic signaling pathway in HF is pivotal. Briefly, human cardiomyocytes express 3 β-receptors with distinct biological effects. Both *β*_1_ and *β*_2_ (normal ratio: 70/30) increase contractility and chronotropy, whereas *β*_3_ receptors act as a counter-regulator, with negative inotropic effects. Essential physiological and pathophysiological responses to beta-adrenergic stimulation are outlined in Figure 1. Numerous studies have revealed some alterations in the beta-receptor system in HFrEF that leads to adverse signal transduction and further deterioration of cardiac function over time (7).

This increase in adrenergic-drive in failing hearts is the fundamental basis for antiadrenergic therapies. In the mid 1970s, pioneers Waagstein and colleagues first demonstrated that BB were safe and could improve clinical status in HFrEF (8). Subsequently, several randomized trials have definitively confirmed that BB therapy significantly reduces mortality in these patients (2–4).

**Figure 2:**
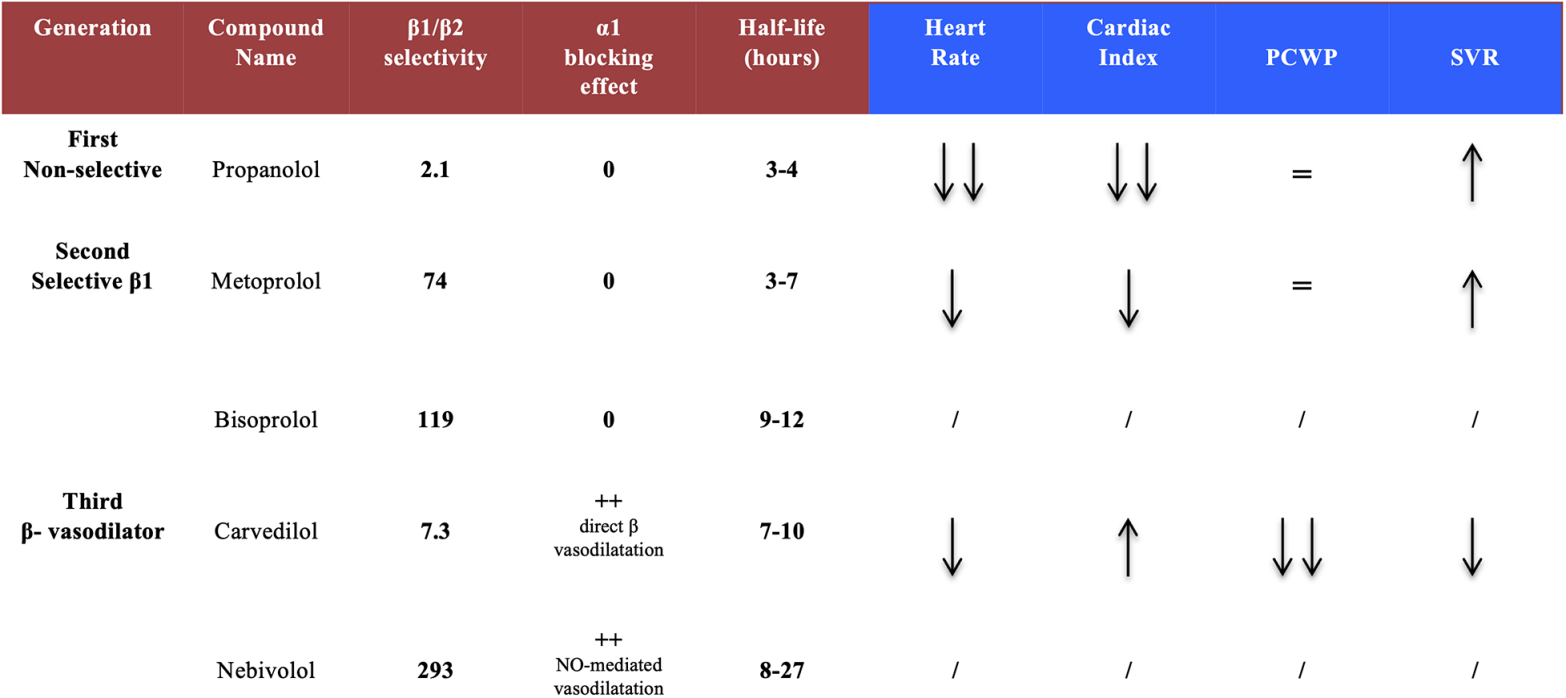
Pharmacological properties of the different beta-blocker generations. Second and third generations show a greater *β*_1_ selectivity and, for third generation compounds, an additional vasodilatory activity. Acute hemodynamic effects reveal a safer profile with carvedilol (as compared to others) regarding cardiac index and systemic vascular resistances. PCWP, pulmonary capillary wedge pressure; SVR, systemic vascular resistance.

Three BB classes can be divided into 2 groups: non-selective and selective compounds. The most important differences are beta-selectivity and ancillary effects. Adrenergic receptor-blocking profiles of the most widely used agents are summarized in Figure 2. Non-selective, first-generation agents have equal affinities for *β*_1_ and *β*_2_ receptors. These agents may be poorly tolerated in patients with severe HF, due to the synergistic actions of blocking both *β*_1_ (negative inotropic effects) and *β*_2_ receptors (rise in peripheral resistance). They cause a marked reduction in heart rate (HR) and an increase in systemic vascular resistance (SVR), which may result in a significant reduction of cardiac output (CO). However, these agents have no significant effect on the pulmonary capillary wedge pressure (PCWP) (9). In contrast, the second-generation agents, such as bisoprolol or metoprolol, are selective (“cardio-selective”) for *β*_1_ receptors and do not block *β*_2_ receptors. Thus, there is little or no increase in SVR. They may reduce cardiac index (CI) to a lesser extent than first-generation agents, and do not affect PCWP. Finally, third-generation agents (carvedilol, bucindolol) can be either selective or non-selective and are distinguished by their vasodilatory effects. This class of agents can provide a more complete anti-adrenergic activity than the second-generation drugs and have a potential advantage because they counteract myocardial depression through their vasodilatory properties and their ability to reduce SVR, which could increase CI and reduce PCWP.

Despite their acute negative hemodynamic effects, BB can induce an improvement of LV ejection fraction (LVEF) and are associated with a dose-dependent impact on reverse LV remodeling (10). The long-term beneficial effects of BB therapy in HFrEF have not been fully elucidated but are thought to mitigate the detrimental consequences of adrenergic overstimulation, resensitize the beta-receptor system and prevent any deleterious signaling effects induced by a prolonged beta-stimulation (11).

Several differences between *β*_1_-selective compounds may have an impact on clinical outcomes; however, at present, there is no strong evidence to favor one drug over another in HFrEF in daily practice, including in acute decompensated situations (12). In patients at highest risk (severely depressed LVEF, congestion, previous need for inotropic support), carvedilol did demonstrate however a mortality benefit within 8 weeks after randomization (13).

## BB management in patients with AHF: a crucial issue to improve outcomes

Despite evidence of efficacy in stable outpatients with HFrEF, BB are underused and at suboptimal doses (14). This clinical inertia is responsible of worse outcomes and quality of life, more symptoms and hospitalizations for HF (15). Data surrounding in-hospital management of HFrEF medications remains very sparse but are of paramount importance. Every possible effort must be made during the hospital course to introduce and/or optimize therapies that have proven to extend survival, and especially BB. However, extrapolating data emanating from trials conducted on stable outpatients to hospital settings on a very different population is obviously complex.

**Figure 3:**
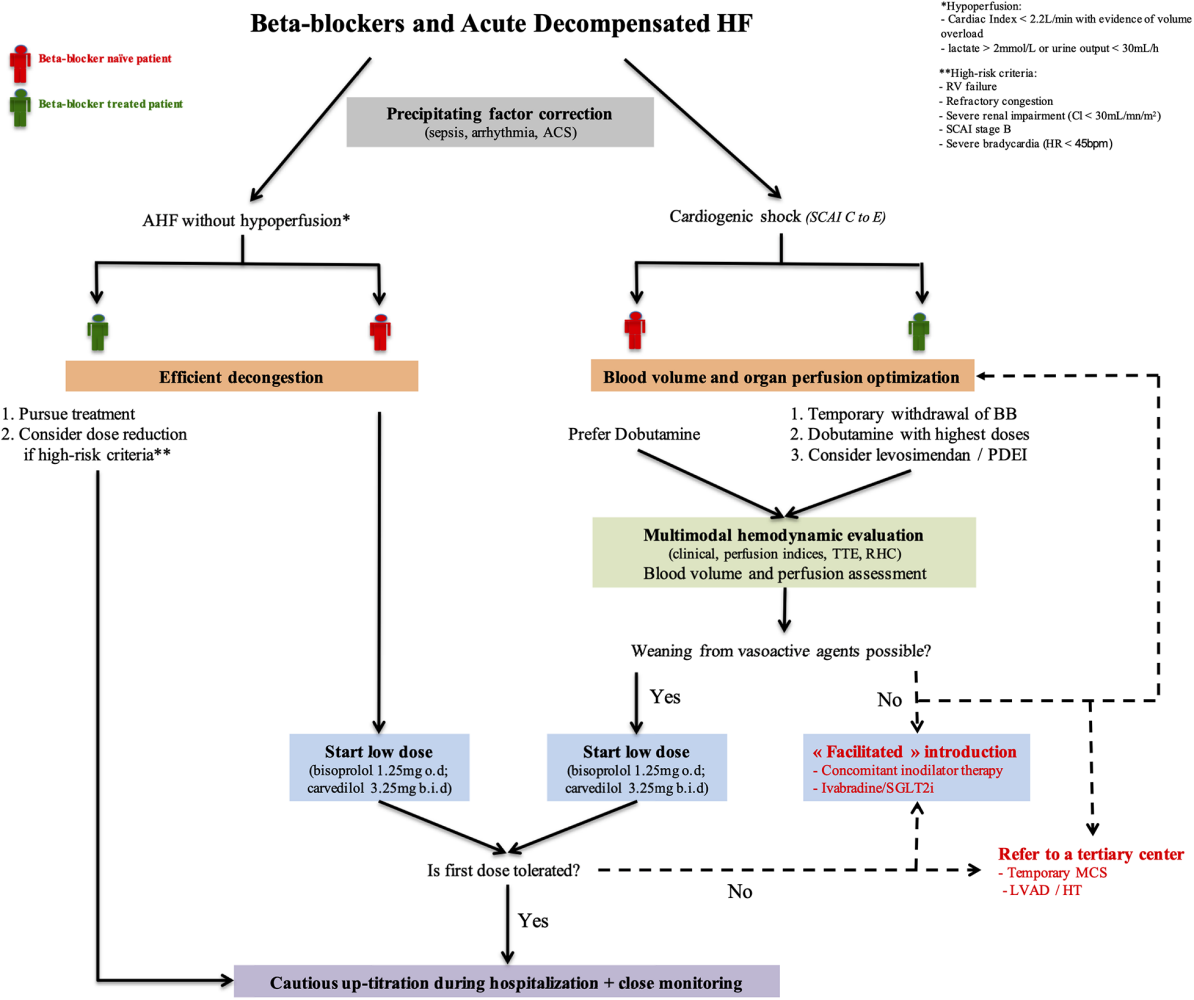
BB management in acute decompensated HF. This schematic overview of BB in AHF highlights the crucial role of accurate patient's hemodynamic condition assessment and its management for clinician decision making. “Facilitated” initiation strategies may be considered for the most severe patients. ACS, acute coronary syndrome; BB, beta-blocker; HF, heart failure; HT, heart transplantation; LVAD, left ventricular assist device; MCS, mechanical circulatory support; PDEI, phosphodiesterase inhibitor; RHC, right heart catheterization; RV, right ventricle; SCAI, Society of cardiovascular angiography and intervention; SGLT2I, sodium-glucose cotransporter 2 inhibitors; TTE, transthoracic echocardiography.

Indeed, therapeutic approaches are highly dependent on AHF phenotype. Based on a pragmatic clinical assessment (initially described by Forrester and Waters), Nohria et al. proposed a classification of AHF patients according to evidence of congestion (wet/dry) and perfusion (cold/warm) (16, 17). This profiling identified significant differences in mortality and re-hospitalizations between groups with the worst outcomes observed in patients with hypoperfusion. Decisions regarding BB continuation, withdrawal, initiation, dose escalation, and dose reduction must consider these differences in hemodynamic profiles (Figure 3).

## Management of BB therapy according to AHF presentation

### Situation N°1: AHF patients without hypoperfusion

#### Available data

Among naïve patients, randomized data demonstrated that BB introduction prior to discharge increases the likelihood that they will be treated and meet the recommended doses at 60 days (5). It further significantly reduced future hospitalizations and HF related death (18). Patients with severe HF have a highly activated sympathetic nervous system and one could argue that BB would be a matter of concern. However, the COPERNICUS (Carvedilol Prospective Randomized Cumulating Survival) and MERIT-HF (Metoprolol CR/XL Randomized Intervention Trial in Congestive Heart Failure) trials showed overall that first, early in-hospital initiation was well tolerated with minimal side effects and second, clinical benefit observed within the first weeks of treatment was similar to that observed in long-term trials (13, 19).

A recent meta-analysis combining observational studies and one small randomized trial suggested that, in previously treated patients, maintaining BB was associated with lower mortality and re-hospitalization rates compared to those that had withdrawn from or never initiated BB therapy (20). Noteworthy, in a subgroup post-hoc analysis from COMET (Carvedilol or Metoprolol European Trial), dose reduction after HF hospitalization was also associated with worse outcomes (21). Acknowledging the fact that BB reduction or withdrawal is associated with more severe forms of HF, these results persisted after adjustment and highlighted the importance of BB continuation for these patients.

#### Current recommendations

The 2021 ESC Task Force on diagnosis and management of HF suggest that evidence based OMT (including BB) have to be pursued in previously treated patients who have experienced worsening HF without hemodynamic instability. Based on current evidence, BB continuation is strongly recommended to avoid hesitancy for re-introduction and to encourage dose escalation during the hospital course. The guidelines also advocate introduction before hospital discharge in naïve patients (class I) once they are considered stabilized (1).

#### Gap of evidence, expert opinion, and up-dated recommendations

Although without hypoperfusion, some high-risk patients may require BB dose reduction or even discontinuation. However, no data nor recommendations clearly indicate to date which patients should be targeted for such strategy. We propose that dose reduction (or discontinuation) should only be restricted to patients with refractory congestion, severe bradycardia, hemodynamic instability without hypoperfusion, severe right ventricle (RV) failure, or severe renal impairment (Figure 3).

Regarding BB initiation in naïve patients, we propose that early in-hospital introduction should be strongly and rapidly considered in all patients with HFrEF without hemodynamic instability, regardless of HF severity. Nevertheless, practical guidelines to introduce BB early and safely during the hospital course are lacking. Our pragmatic guidance for a successful introduction is exposed below.

### Situation N°2: AHF patients with hypoperfusion

#### Available data

In patients presenting with hemodynamic compromise, the first interrogation is to what extent BB therapy could be maintained or on the contrary should be temporarily withdrawn. Discontinuation of BB in patients requiring inotropic support may expose them to a possible rebound increase of myocardial ischemia or severe arrhythmias that would lead to worse outcomes. Two post-hoc analyses of trials suggested a deleterious effect of BB withdrawal in patients receiving milrinone, dobutamine or levosimendan (22, 23). However, ischemia and arrhythmia-related events were unfortunately not reported. More importantly, eligibility to a vasoactive therapy was mostly led to the attending physician feeling basis rather than on objective indices of tissue hypoperfusion in these 2 studies. For CS patients, a retrospective French study and a subgroup analysis of the DOREMI (Dobutamine Compared to Milrinone) showed that BB use on hospital admission or 24 h before randomization led to a lower mortality, although it was not mentioned whether BB were withdrawn, reduced or not throughout hospitalization (24, 25).

Second, hemodynamic responses of most commonly used inotropic agents may vary in patients previously treated with BB and could influence physicians' choice of one drug over another to restore adequate perfusion. Dobutamine is a catecholamine with *β*_1_ and *β*_2_ agonist activities, but the other drugs (milrinone, enoximone or levosimendan) do not stimulate *β* receptors to drive contractility. Their inotropic effects increase CO, partly by lowering SVR and left ventricle end-diastolic pressure (LVEDP), without excessively changing HR. To date, no study has demonstrated a clinically relevant difference in the improvement of hemodynamic parameters between dobutamine and other inodilators. Randomized trials have shown that effects of levosimendan, unlike those of dobutamine, were not affected by concomitant use of BB (26), whereas negligible differences were observed between milrinone and dobutamine (25). Regarding outcomes, conflicting results were produced when hemodynamic parameters were translated into clinical benefits. Compared to dobutamine, levosimendan has shown a potential benefit in reducing all-cause mortality in patients with AHF taking BB in a post-hoc analysis of the SURVIVE Study (27). This benefit has not been observed with milrinone in a post-hoc analysis of the DOREMI study (25). Interestingly, none of those studies listed the type and doses of BB used, which is a limitation for drawing firm conclusions.

Of note, pharmacological properties of different generations of BB significantly impact hemodynamics, when combined with a vasoactive agent. Administering dobutamine in patients previously exposed to metoprolol tartrate or carvedilol markedly attenuated its hemodynamic effects, especially with carvedilol. Conversely, BB (with either metoprolol or carvedilol) did not alter the hemodynamic improvements achieved with enoximone (28). These results highlighted the potential role of phosphodiesterase (PDE) inhibition in unstable patients that were already taking BB, particularly carvedilol. To the best of our knowledge, no study has compared different types of BB with concomitant use of milrinone or levosimendan.

### Current recommendations

* *There are to date no recommendation on how BB should be managed in these patients with hypoperfusion. In addition, no clear recommendations are available on which inodilators should be used in patients under chronic BB therapy at admission for CS.

#### Gap of evidence, expert opinion, and up-dated recommendations

Based on the pathophysiological considerations and the negative inotropic effects of BB and because clinical data are very limited, we think it is safer to systematically withdraw BB therapy in case of CS stage C–E of the Society of Cardiovascular Angiography and Intervention (SCAI) classification (29). In the highly specific situation of CS triggered by recurrent ventricular arrythmias, although BB may have a potential interest to control cardiac rhythm and although abrupt discontinuation may lead to a potential rebound effect, we think that using BB is still hazardous and we propose to prefer amiodarone and lidocaine in this setting (pursuing BB should be restricted to highly selected cases). To our opinion, hemodynamic instability without hypoperfusion (SCAI shock stage B, referred as beginning CS/compensated shock/pre-shock) should not be a trigger for systematic BB discontinuation (dose reduction can be considered).

Dobutamine remains the first line inodilator to normalize hemodynamics in patients with CS, especially for BB naïve patients. Inotropic effects of dobutamine are highly dependent on beta-receptors saturation so highest doses may be used in patients previously treated with BB. We propose to consider levosimendan or PDE inhibitors in patients under chronic BB therapy and in the case of dobutamine failure (second line therapies) (Figure 3). However, this relies on scarce data with no proven mortality benefit. Non-adrenergic inodilators are of potential interest, but physicians must embed the pharmacological properties of previous BB use (i.e., selectivity, vasodilatory effects) in addition to the patient's hemodynamic status, potential side effects (arrhythmias, hypotension), and costs of these drugs. PDE inhibition seems however to be an attractive alternative with carvedilol.

Whether to introduce (or restart) BB in patients with recent hypoperfusion remains a matter of debate. A recent large multicenter registry of patients with CS (30) has revealed that, for about half of the admitted patients, shock was the first manifestation of their disease. This finding implied that most patients were not treated with any guidelines-directed medications at admission. The timing of BB (re-)introduction is of paramount importance; it requires careful evaluation and close monitoring to ensure optimal safety (see dedicated paragraph below). Once clinical stabilization occurs (i.e., congestion and perfusion are controlled, see below), we suggest that BB have to be cautiously resumed as soon as possible, usually after at least a short period of weaning from vasoactive drugs (usually 24 h).

## Practical considerations for BB initiation

### General principles

**Figure 4:**
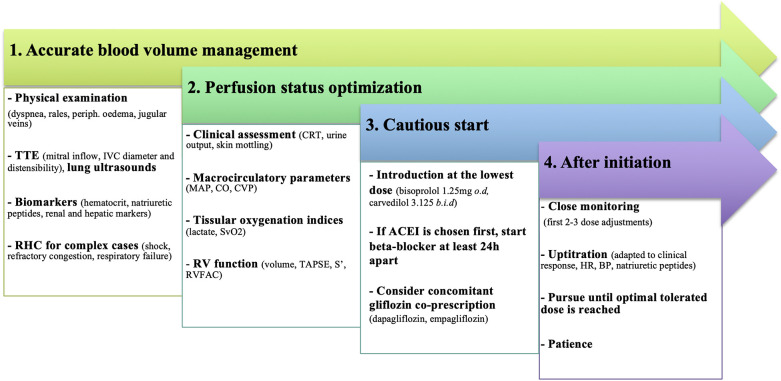
Practical considerations for BB introduction in patients with AHF. To minimize treatment failure and improve tolerance, introduction should only be considered after a multi-parameter evaluation, especially for congestion and perfusion status. ACEI, angiotensine converting enzyme inhibitor; BP, blood pressure; CO, cardiac output; CRT, capillary refill time; CVP, central venous pressure; HR, heart rate; IVC, inferior vena cava; MAP, mean arterial pressure; RHC, right heart catheterization; RV, right ventricle; RVFAC, right ventricular fractional area change; TAPSE, tricuspid annular plane systolic excursion; TTE, transthoracic echocardiography.

BB treatment should always be started cautiously at the lowest dose. A careful, step-by-step, personalized approach is recommended, where all available data on symptoms, clinical signs, hemodynamics, and biological parameters are integrated at each time-point. Figure 4 outlines the practical considerations that should be taken when introducing BB in patients with AHF.

Any condition that may compromise treatment tolerance should be carefully addressed. Initiating BB in unstable conditions will result in variable degrees of HF worsening, ranging from congestion to new shock development and stressing the need for close monitoring. In addition to factors that can jeopardize a previously stable condition (sepsis, arrhythmias, or acute coronary syndromes), the most common factor is an inaccurate hemodynamic evaluation. Clinicians must carefully assess inadequate decongestion, persistent clinical/biological hypoperfusion, and misestimation of RV dysfunction through a multimodal evaluation.

### Filling pressures and blood volume management

The first consideration is to achieve “complete” decongestion. Despite its poor reproducibility, physicians must first rely on their physical examination and identify any signs of dyspnea or orthopnea, pulmonary rales, ascites, or peripheral edema. Well-recognized and readily monitored indicators of congestion are represented by hematocrit, natriuretic peptides, and renal or liver function markers. Attentive bedside trans-thoracic echocardiography (TTE) is an effective tool to estimate LVEDP, whereas lung ultrasound is widely accepted and routinely used for evaluating pulmonary edema (31). Finally, for the most complex AHF patients (i.e., unresponsive to initial therapy, severe biventricular impairment, shock, respiratory failure), right heart catheterization (RHC) remains the gold standard for assessing both right and LV filling pressures and should be strongly considered.

### Perfusion status

BB should be started in hemodynamically optimized patients in the absence of symptomatic bradycardia. Hypotension is associated with increased mortality in AHF and usually represents a relative contraindication to BB introduction. However, classical macro-circulatory endpoints such as HR, blood pressure (BP), CO and central venous pressure (CVP) could be dissociated from tissular perfusion. Successful resuscitation and, by extension, implementation of BB should preferably be based on a tissue-perfusion approach to avoid treatment failure. Hypoperfusion is indeed a specific feature of shock, even in the absence of hypotension; thus, it must be promptly identified. Common physical signs of hypoperfusion (e.g., capillary refill time, skin mottling, confusion) are important warnings but are not a substitute for an accurate multimodal assessment which requires measurements of biological perfusion indices (central venous oxygen saturation, lactate, venoarterial CO_2_ pressure gradient, and combinations of these), TTE evaluation and, in complex situations, use of pressure and impedance-based techniques.

### Right ventricle function evaluation

In chronic HF, RV failure is an independent predictor of survival. In addition, the Beta-Blocker Evaluation of Survival Trial (BEST) showed that, in patients with an RV ejection fraction below 20%, BB therapy with bucindolol was associated with worse survival (32). The reasons for these poor outcomes remain unclear and it should be noted that none of the landmark BB trials have reported RV ejection fraction at baseline (2–4). A careful evaluation of RV function is advised, but this evaluation is highly dependent on load conditions. Thus, the load should ideally be optimized before making RV assessment. Severe RV dysfunction could probably influence the choice of BB; a preferable choice would be an agent without vasodilatory effects, but this statement is purely speculative and based on pathophysiological data. Moreover, severe RV dysfunction renders RV output rate-dependent so that negative chronotropic effect of BB could be harmful. Careful attention is required among patients with compensatory tachycardia to avoid a drastic drop in stroke volume. Thus, initiation and titration have to be very cautious in this subset of patients and should probably be postponed in cases of overt severe RV failure.

### How to improve successful initiation: ivabradine, inotropic support, ultra-short acting beta-blockers, and digoxin

Ivabradine is a selective I_f_ channel inhibitor that reduces HR without affecting myocardial contractility. It can increase both CO and BP, reduces afterload and improves adverse LV remodeling (33). Ivabradine may result in improved BB tolerance in CS patients, but solid data are lacking. Preliminary studies appeared promising and reported a significant improvement in hemodynamic parameters, which permitted weaning from dobutamine and successful BB initiation (34).

Requirement of inotrope therapy is one of the main exclusion criteria in most randomized trials that have demonstrated mortality benefits of BB (2–4). However, when BB are initiated with inotropic support, it seems better tolerated. First, BB might attenuate the pro-arrhythmic effects of these drugs and second, vasoactive agents (particularly those with activities beyond beta-receptor stimulation) are expected to preserve their inotropic properties, even in the presence of BB therapy. The combination could promote the advantages of BB and cancel out their harmful properties, thus overcoming short-term intolerance and facilitating up-titration (35–37.) Some observational data have highlighted a potential role for enoximone in patients with dobutamine-dependent HF and in patients who have been weaned off dobutamine but have failed to initiate BB. These results underscored the impact that early administration of HF therapies could have, even in the most critical conditions (38).

The benefits of ultra-short acting BB are inconclusive. Due to their attractive pharmacologic profiles, especially for landiolol (which is *β*1 selective), impact on cardiac function would be minimal (39). Landiolol might be useful as a “tolerance test” or a bridge for usual recommended oral BB. However, the precise hemodynamic response remains unclear in the setting of severe HFrEF and landiolol administration should be limited to unstable situations, where rapid control of supra-ventricular arrhythmias is needed and is suspected to be responsible for LV dysfunction (40).

Finally, the use of digoxin is a potential area of interest. Digoxin has indeed inotropic effects that could offset the detrimental properties of BB at initiation. Nevertheless, the benefit of that approach currently remains hypothetical. Digoxin may have a specific place in patients with atrial fibrillation (AF).

All these approaches are to date speculative (no robust data) and should be restricted to patients with a previous BB introduction failure. It is very difficult to favor one approach over another and to target pragmatic criteria to individualize the choice in daily practice.

### BB responsiveness and titration

The action of BB may be biphasic, and patients should be informed of potential adverse effects (excessive HR and BP reduction, fluid overload) during initial dose adjustments. Titration must initially be cautious and tailored to the clinical response. Natriuretic peptides levels often parallel the benefits of HF therapies and lower concentrations after treatment introduction not only reflect responders but also represent a signature of LV structural changes and reverse remodeling (41). Once AHF patients are stabilized, the recent “The Safety, Tolerability and Efficacy of Rapid Optimization, Helped by NT-proBNP Testing, of Heart Failure Therapies” (STRONG-HF) study has revealed that an intensive strategy of HF therapies (including BB) initiation before hospital discharge with a rapid up-titration within the first 6 weeks was safe and reduced 180-day all-cause mortality and re-hospitalization for HF (42).

### Should other HF therapies be considered before attempting BB introduction?

There is no significant interaction between HFrEF medical therapies with respect to efficacy, supporting the evidence that each class acts on a distinct pathophysiological substrate. Therefore, a clear-cut optimal sequencing has not yet been defined. In stable patients, guidelines advise that all drugs can be started together (1). BB used as the first neurohormonal antagonist is safe (43), and regardless of background therapy. By contrast, in unstable patients, particularly in those with resolved hypoperfusion, it may be safer to start BB and angiotensin converting enzyme inhibitors at least 24 h apart. Potential HF worsening following BB introduction can be mitigated by early concomitant initiation of sodium-glucose cotransporter 2 inhibitors and represents a potential era of future investigation.

## AHF and AF

Prevalence of AF increases with HF severity and the question of whether BB are associated with a better prognosis in this subgroup of patients has recently been raised (44). In stable patients, it seems that BB usefulness goes far beyond HR control. In AHF patients however, AF may be the cause or the consequence of decompensation and it is often not easy to distinguish both situations in daily practice. In the former, BB may be used very early (especially ultra-short acting agents) when in the latter they should be introduced very cautiously only after stabilization.

## BB for HF with midrange and preserved EF

Evidences that BB benefits in HF with EF ≥40% are suggestive. It is unclear whether adrenergic activation has a similar impact as compared to HFrEF and available data are inconclusive regarding mortality benefits (45). However, a previous meta-analysis including the “Randomized trial to determine the effect of nebivolol on mortality and cardiovascular hospital admission in elderly patients with heart failure” (SENIORS) study has suggested similar effects in reduction of mortality with BB use in HFrEF and also in HF with midrange EF (HFmrEF) patients (46, 47). Therefore, BB therapy might be considered in HFmrEF (class IIb recommendation) but is not recommended for HF with preserved EF unless a secondary indication exists (i.e., AMI, arrhythmias) (1, 48).

## Conclusions and future perspectives

For over 20 years, BB have been the cornerstone therapy for stable HFrEF. However, its management in the setting of AHF remains challenging. The different approaches are highly dependent on patient's clinical phenotype as well as the existence of prior BB therapy. We aimed to provide a concise algorithm based on these two major considerations.

Paucity of data on facilitated strategies to improve BB initiation prompt us to conduct largescale prospective cohort studies that could delineate their role before being widely embraced in practice. Future trials may help to clarify some points in the future. Most relevant questions would be to assess the possibility to pursue or not BB in patients with a “pre-shock” condition (SCAI stage B), to test which inodilators should be preferred in patients with classic CS and under chronic BB therapy before admission, and finally to compare strategies of facilitated initiation of BB vs. usual low-dose BB initiation. Finally, although commonly accepted, pursuing BB therapy at the same dose in patients with AHF and without hypoperfusion has never been specifically tested in dedicated randomized trials. This frequent clinical situation also deserves more insights to our opinion.

Of note, when attempts to introduce BB fail after careful attention and correction for residual confounders, patients that remain truly intolerant have to be referred to a tertiary center for potential durable mechanical circulatory support or to determine eligibility for heart transplantation.
